# Dendritic Branch Intersections Are Structurally Regulated Targets for Efficient Axonal Wiring and Synaptic Clustering

**DOI:** 10.1371/journal.pone.0082083

**Published:** 2013-12-13

**Authors:** Monika Pinchas, Danny Baranes

**Affiliations:** 1 Department of Electrical and Electronics Engineering, Ariel University, Ariel, Israel; 2 Department of Molecular Biology, Ariel University, Ariel, Israel; Tel Aviv University, Israel

## Abstract

Synaptic clustering on dendritic branches enhances plasticity, input integration and neuronal firing. However, the mechanisms guiding axons to cluster synapses at appropriate sites along dendritic branches are poorly understood. We searched for such a mechanism by investigating the structural overlap between dendritic branches and axons in a simplified model of neuronal networks - the hippocampal cell culture. Using newly developed software, we converted images of meshes of overlapping axonal and dendrites into topological maps of intersections, enabling quantitative study of overlapping neuritic geometry at the resolution of single dendritic branch-to-branch and axon-to-branch crossings. Among dendro-dendritic crossing configurations, it was revealed that the orientations through which dendritic branches cross is a regulated attribute. While crossing angle distribution among branches thinner than 1 µm appeared to be random, dendritic branches 1 µm or wider showed a preference for crossing each other at angle ranges of either 50°–70° or 80°–90°. It was then found that the dendro-dendritic crossings themselves, as well as their selective angles, both affected the path of axonal growth. Axons displayed 4 fold stronger tendency to traverse within 2 µm of dendro-dendritic intersections than at farther distances, probably to minimize wiring length. Moreover, almost 70% of the 50°–70° dendro-denritic crossings were traversed by axons from the obtuse angle’s zone, whereas only 15% traversed through the acute angle’s zone. By contrast, axons showed no orientation restriction when traversing 80°–90° crossings. When such traverse behavior was repeated by many axons, they converged in the vicinity of dendro-dendritic intersections, thereby clustering their synaptic connections. Thus, the vicinity of dendritic branch-to-branch crossings appears to be a regulated structure used by axons as a target for efficient wiring and as a preferred site for synaptic clustering. This synaptic clustering mechanism may enhance synaptic co-activity and plasticity.

## Introduction

Dendrite morphology is important for determining what signals a neuron receives and how these signals are integrated. However, a major unresolved question is whether dendritic morphology can predict functional synaptic connectivity. One potential influence on synaptic input distribution may be the spatial pattern of dendritic branches within dendritic trees, as well as the relative arrangement of neighboring trees. Overlap of dendritic trees was shown to be a regulated phenomenon, as evinced by specific neuron populations found to innervate targets with substantial territorial overlap [1], and by cellular and molecular cues regulating the spatial arrangements of dendritic branches within and between arbors [2]. For instance, adhesive interactions between arbors can stabilize dendritic branches at specific configurations [3], bundle those branches and possibly coordinate their activity [4]. The advantage of such a controlled design of arborization is the minimization of the path length from the dendritic root to each of its synaptic inputs, thus constraining the total length of wiring [5]. This same logic appears to be followed by innervating axons which may choose routes along specific dendritic morphologies to minimize wiring lengths of both axons and dendrites. Therefore, understanding how dendritic branches are patterned relative to one another can help to uncover the functional logic of neural circuit organization.

One parameter of dendritic structure potentially involved in the minimization of neuronal circuit wiring cost is the clustering of synaptic inputs along dendritic branches [6], [7]. The clustering of the synaptic connections has a functional meaning at several levels. First, superlinear integration of clustered synaptic inputs can significantly increase the computational power of neurons [8]–[10]. Second, the simultaneous activation of clustered synapses influences neuronal firing more strongly than does the firing of disperse synapses [6], [8], [11]–[13]. Third, the grouping of synapses along individual dendritic branches enhances synaptic plasticity and may consolidate information storage [14]–[19], making the branches, rather than individual synapses, the primary functional units for long-term memory function. However, it is largely unknown how dendritic branches are innervated by axons, or what rules determine their connectivity patterns and consequent synaptic clustering [20]. It was suggested by several studies that synaptic clustering is related to the activity of the contacting neurons. For example, correlated activity at the site of synaptic clustering may lead to synaptic clustering [21]. It is also possible that clustered synaptic organization is established through local plasticity [16], [18] or by experience [22]. Other works suggested that synaptic clustering occurs by convergence of functionally related axons onto dendritic branches that correlate with their activity [9], [14], [15], or that clustering is the outcome of localized dendritic signaling mechanisms [23], such as local spread of Ras activity [24].

However, in contrast to the above, there is evidence that synaptic allocation may be organized anatomically, without the involvement of neuronal activation. In spinal circuits controlling swimming in hatchling frog tadpoles, the probability of contact between axons and dendrites could be predicted simply by their anatomical overlap [25]. It was thus suggested that axo-dendritic contacts are determined by the geography of the spinal cord, primarily by the dorso-ventral distributions of axons and dendrites. Similarly, Hill et al [26] established a simulation that predicted neural circuitry generation in the neocortex by random overlap of dendritic and axonal trees. Recently, Packer et al demonstrated that the connectivity maps of interneuron contacts could result from the overlap of axonal and dendritic arborizations [27]. According to the above studies, synaptic clusters may arise from axons arbitrarily found in close proximity to dendritic branch crossings. However, such a random clustering mechanism is difficult to accept as it lacks regulation and thus may generate synaptic clustering at low frequencies.

Alternatively, regulated axonal convergence, such as that which can be induced and tuned, is more appropriate for the control of a fundamental neuronal asset such as synaptic clusters. For example, in olfactory glomeruli, structures enriched with synaptic clusters, converging axons are abundant and their behavior is regulated. Their convergence can be stimulated by semaphoring 1a [28], mediated through a G protein/cAMP signaling cascade [29], whose feedback loop is controlled by the retinoic acid receptor and CNGA2 channel signaling [30]. Also, their convergence may also derive from the axonal tendency to contact targets while using a minimal extension length [31], as mentioned above. This “minimal length” principle of wiring efficiency may drive axons to contact as many target dendrites by the shortest path possible. It is likely that dendrites organize their structure in order to provide axons with efficient wiring targets exhibiting high branch-to-branch proximity, as seen in sites of branch crossings. A similar solution is seen in cortial map formation where there is an evolutionary pressure to place connected neurons as close to each other as possible to innervate distant neurons with minimal axonal length.

In the present study, axonal convergence near branch-to-branch crossing sites was a favored wiring behavior of axons that produced synaptic clusters in hippocampal cultures. These results accumulated through a computerized assay we developed to quantify the overlap between crossing dendritic branches and axons. Analysis of meshes formed by hundreds of interacting neuritic segments revealed that the angles through which dendritic branches cross each other are under regulation and they affect the orientation through which axons approach the crossing sites. It was also found that the dendritic branch intersections vicinity is a preferable target for axonal convergence and synaptic clustering. The relevance of this axonal convergence-dependent clustering mechanism to neuronal wiring and synaptic co-activity is discussed.

## Methods

### Ethics Statement

This study was carried out in strict accordance with the recommendations in the Guide for the Care and Use of the Board of Animal Experiments of the Israeli Ministry of Health. The protocol was approved by the Committee of the Ethics of Animal Experiments of the Ariel University (Permit Number: IL-36-05-12).

### Cell Culture

Hippocampal Dentate Gyrus-CA3 regions were dissected out from brains of P1–P4 Sprague Dawley rat pups, as described previously [32]–[34]. Briefly, the tissue was treated for 30 min at 37°C with 0.25% trypsin (Sigma, type XI); dissociated gently and plated at a concentration of 2×10^5^ cells/ml onto 12 mm glass cover slips coated with poly-D-lysine (Sigma, 20 µg/ml) and laminin (Collaborative Research, 10 µg/ml). Cells were plated in MEM (Sigma) containing 10% heat inactivated normal goat serum, 1% L-glutamine and 0.8% D-glucose. One day after plating, cells were transferred to serum-free medium containing 45% MEM, 40% DMEM, 10% F12, 0.25% (w/v) BSA, 1% DiPorzio supplement [35], 0.34% D-glucose, 0.5% B27 supplement, 0.25% L-glutamine, 0.01% kinurenic acid, and 0.01% of mixed 70% uridine and 30% fluoro-deoxy-uridine. The cultures were maintained for up to 3 weeks in a 37°C humidified incubator with 5% carbon dioxide.

### Immunocytochemistry

Cells were stained as described [33]. Briefly, cells were fixed for 10 min at room temperature with 4% paraformaldehyde (PFA), permeabilized with 0.25% Triton, and blocked with 3% normal goat serum. The cells were then incubated overnight at 4°C with a mouse monoclonal anti-microtubule associated protein 2 (MAP2) antibody (1 µg/ml, Sigma) and a rabbit polyclonal anti neurofilament M (NFM) antibody (0.5 µg/ml, Millipore). Cells were then washed and incubated for 1 h at room temperature with secondary antibodies conjugated to Alexa-488 or Cy3 (2 µg/ml, Invitrogen), washed and mounted on slides with the anti-quenching agent 1,4-diazabicyclo[2.2.2]octane (2.5%, Sigma).

### FM1-43 Labeling of Active Synaptic Contacts

Uptake and secretion of FM1-43 were monitored as described [32]. Briefly, 12 day old cultures were exposed for 30 s to 15 µM FM1-43 (Invitrogen) in Tyrode’s buffer (in mM: NaCl 119; KCl 5, CaCl2 4, MgCl2, 2; glucose 30, HEPES 20; pH. 7.3), supplemented with 45 mM K^+^ followed by a 3-min wash with Tyrode’s buffer at a rate of 1 ml/min. Following image acquisition, FM1-43 was secreted in response to 45 mMK^+^ in Tyrode’s buffer for 30 s, followed by a 3-min wash with Tyrode’s buffer at a rate of 1 ml/min. Images were obtained and subtracted from uptake images of the same field. The result was considered proportionate to the size of the releasable vesicle pool.

### Microscopy

Stained cultures were visualized through a Zeiss Axio Observer.Z1 Microscope equipped with the following objectives: Plan-Neofluar 10×/0.30, Plan-Neofluar 25×/0.8 Imm Corr W/Gly/Oil, Plan-Neofluar 40×/1.30 Oil and Plan-Apochromat 63×/1.40 Oil. Images were captured with a 24.57-MHz CCD camera (AxioCam MRm rev.3, Zeiss) operated by the Zen 2010 software.

### Image Analysis

Photoshop Cs6 version (Adobe Systems Inc.) was used for image processing and for manual marking of dendritic crossings and their connecting dendritic segments. Manual measurement of lengths and angles was performed with the ImageJ software (NIH). The CCM software was developed using Matlab R2012a.

### General Description of CCM Operation

CCM creates a connectivity map of neurite intersections through the following principles:

- identifying pixels located on neurites (“positive pixels”), based on the difference between their gray value and that of the background.
- identifying neurite intersections, based on the intersections’ greater thickness than that of the neuritic shaft.
- identifying intersection to intersection connecting neurites (“segments”), connected intersections are associated through a continuous row of positive pixels.
- repeating a–c with varying thickness thresholds to identify thinner intersections and segments.

**Figure 1:**
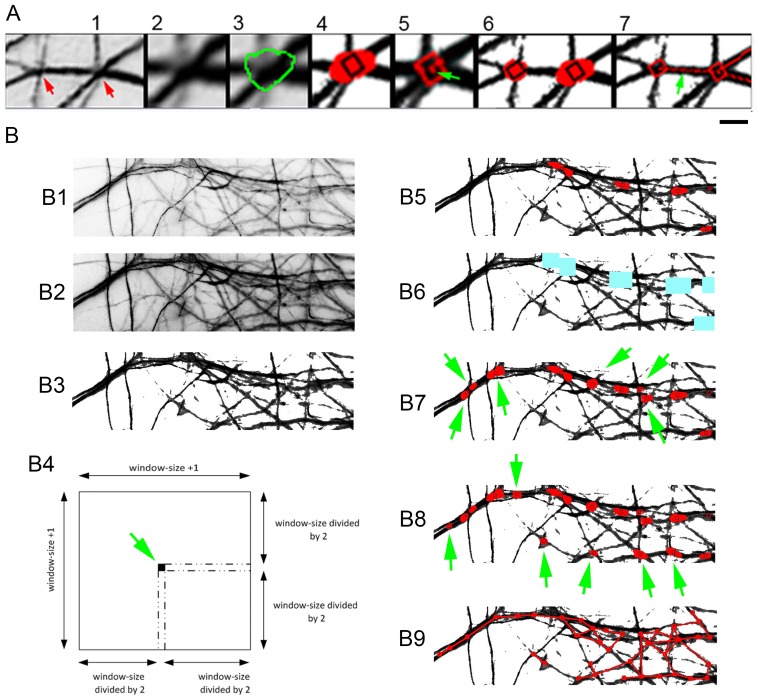
CCM procedure for generating connectivity maps. (A) Basic principles of identification of dendritic branch intersections and their connections. Dendritic intersections (A1) are identified based on their greater width compared to the dendritic segments composing them (A2, labeled in A3). The intersections are marked (A4), centralized (arrow in A5) and connected by a line (A6, arrow in A7). (B) General description of CCM operation. (B1) Starting image – grayscale, inverted. (B2) Contrast optimization. (B3) Background whitening. (B4) A scanning box with centralized pixel (arrow). (B5) Identified junction area (red) with estimated “intersection center pixels” (black squires). (B6) Areas at and near intersections are removed (colored in blue). (B7) second iteration on an updated image (using varying threshold for intersections) reveals thinner intersections (arrows). (B8) A third iteration generated after removal of the B7 new intersections and using a new threshold revealed additional and even thinner intersections (arrows). (B9) Generation of lines connecting the intersections, according to the neuritic map. Scale: A - 3.5 µm; B - 12 µm.

CCM protocol proceeds through the following steps:

- Step 1: Image preparation (Fig. 1B1):
  - CCM uses grayscale images in which dendrites and axons are indicated by pixels of lower gray value than the background. Such images were generated by converting original RGB images to grayscale and inverting them (using PhotoShop). Cell bodies were manually deleted.
- Step 2: Reading the image data:
  - The gray value of each pixel in the image was collected and organized as a two dimensional matrix.
- Step 3: Adjusting the image contrast (Fig. 1B2):
  - The image contrast was adjusted so that all areas relevant for analysis have lower values compared to the background. This adjustment was done by applying a denoising filter (gamma_density).
- Step 4: Unifying all background pixels by converting them to white (Fig. 1B3):
  - Such unification was required to avoid background fluctuations when assigning thresholds. Applied by defining background pixels as below the detection threshold and assigning them white grayscale value (255 in 8bit images).
- Step 5: identification of non-background pixels.
  - The software finds the location (row and column) of each pixel with grayscale value lower than 255.
- Step 6: identification of intersections:
  1. A square box of a configurable size (“box-size”) was positioned on the first intersection center pixel (<255 gray value), so that the pixel was at the center of the box (Fig. 1B4).
  2. The position and location of pixels (<255 gray value) within the box was then registered.
  3. If a particular box contains a number of pixels (<255 gray value) that surpasses a predefined threshold, it was considered to have an intersection.
  4. An estimation technique was used to allocate a single pixel as the center of this intersection. This point was then colored in red.
  5. The box moved to the next pixel (<255 gray value) and repeats stages 6.1–6.4. The outcome of scanning the entire image was the formation of large red points near and around neuritic intersections (Fig. 1B5).
  6. Estimation was then performed specifically on the large red points to assign a single pixel to their center (“intersection center pixel”). These pixels were considered the points of intersection and were marked by small black squares (Fig. 1B5, see also Fig. 1A4 and 2A6).
- Step 7: Generation of connectivity maps of intersections of selective thickness.

The method described in step 6 is a generalized procedure for detecting intersections, regardless of their size. However, due to the large diversity in intersection thickness, it was frequently difficult to detect them both thick and very fine intersections of the same image by using a constant pixel threshold. Consequently, intersections of different thicknesses were scanned separately, using the following procedure:

-
  1. Thick intersections were scanned (with appropriate threshold (step 6.3)) and registered
  2. The intersections detected in 7.1 were removed from the image by positioning a box on the red pixels converting all non-white pixels in the box into white (Fig. 1B6).
  3. The image was updated
  4. A second iteration of a new threshold (of step 6.3) was performed on the updated image to reveal thinner intersections (Fig. 1B7).
  5. Steps 7.2–7.4 were repeated with a new threshold (100 pixel less than in step 6.3), to detect still finer intersections (Fig. 1B8). This procedure was repeated for 6–7 itrations in order to reliably cover almost the entire range of intersection thicknesses.
- Step 8: Detection of connections between intersections.

Connected intersections have a defined amount of positive pixels between their centers. To detect such sets the following procedure was used:

-
  1. A scanning box was positioned on an intersection center pixel (as in 6.1, image type – as Fig. 1B4).
  2. The number of positive pixels within the box was registered
  3. The box was moved along a virtual line connecting the pixel to a second intersection center pixel.
  4. The sum of positive pixel number in all boxes along the line was registered. If this sum was higher than a predefined value then it was said that the two intersection centers are connected.
  5. Stages 8.3–8.4 were repeated for lines connecting the pixel in 8.1 to all other intersection center pixels in the image.
  6. Stages 8.1–8.5 were repeated for each of the intersection center pixels. Connecting lines that were already tested in previous runs were not repeated. The outcome of such operation was a reconstruction of the neuritic network (Fig. 1B9).
- Step 9: Extraction of morphometric parameters:

Reconstruction of the neuritic network enabled the measurement of angles between crossing segments’ angles, length and number.

### Statistics

GraphPad Prism 5.02 (GraphPad Software) was used for statistical analysis. Unpaired two tailed student *t* test (95% confidence interval) was used when double sets of measurements were available. When more than two sets were analyzed, one way ANOVA with Bonferroni’s multiple comparison post test as well as F test were applied. Results are presented as the mean ± SEM.

## Results

### Neighboring Dendrites in Culture Intersect Their Branches in a Directed Fashion

**Figure 2:**
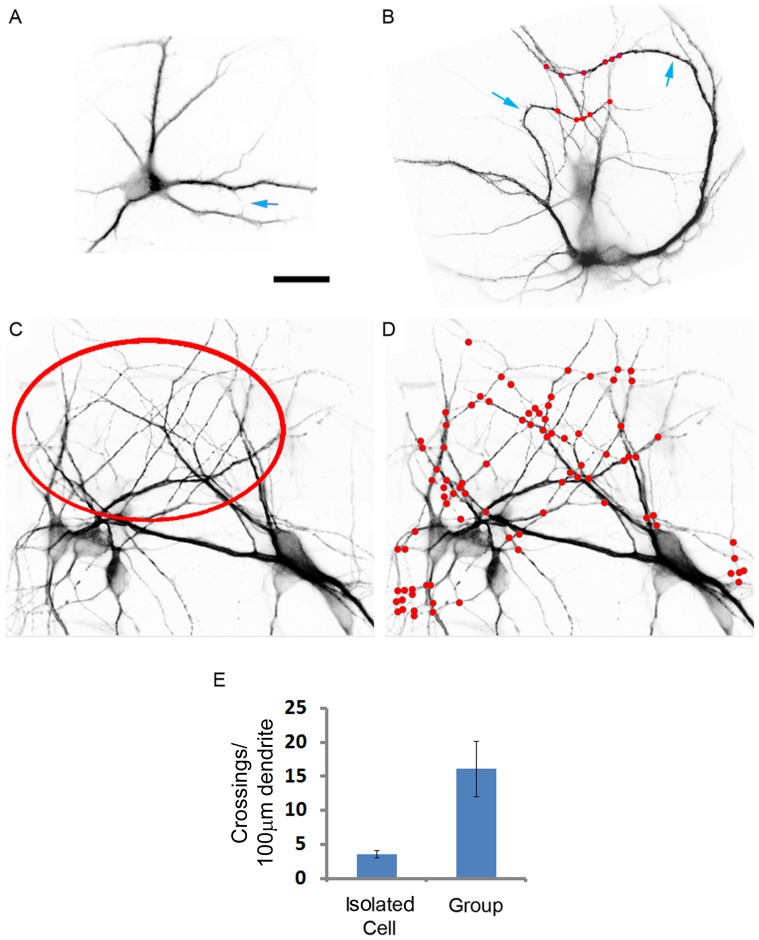
Neighboring dendrites preferentially generate contacts among their branches. Pictures are inverted images of hippocampal neurons grown in culture for 5 days and labeled with anti-MAP2 antibody. (A) An isolated neuron with no physical contacts with other neurons made a single contact between its own branches (arrow). (B) Within a neuronal assembly, dendritic branches turn (arrows) to contact branches of neighboring dendrites (red spots). (C, D) A dense culture in which all neurons orient dendritic branches toward a major overlapping area (circled) where heavy crossing takes place (D, red spots). (E) Dendrite-dendrite contact density is significantly higher among grouped rather than isolated neurons. Shown is the mean ± SEM. Scale: 20 µm.

Whether dendritic trees develop their morphology arbitrarily or in an ordered fashion is an open question. We found indications that in neuronal networks in culture, dendrites ramify in a non-random fashion. Their branch orientation is selected in relation to the orientation of neighboring branches. This phenomenon was discovered by comparing behavior of dendrites in a neuronal assembly to that of dendrites of isolated neurons. Dendrites of neurons growing in complete isolation distributed their branches in an unrestricted range of angles (Fig. 2A). These branches spread away from each other and rarely contacted. By contrast, when dendrites grewin an assembly, a portion of their branches turned from their original growth direction, sometimes more than 90°, and extended toward nearby branches with which they crossed and contacted (Fig. 2B). In dense cultures, this behavior expanded and many of the branches oriented toward an area where they all overlapped (Fig. 2C). Obviously, in such cultures, contact numbers was much higher than in that of the isolated neurons (Fig. 2D). Dendrites in a group had approximately 4 fold more contacts per 100 µm dendrite (Fig. 2E) than did isolated dendrites. Whereas isolated dendrites had 3.6±0.713 (Mean±SEM, n = 12 cells, 119 crossings, total 3367 µm dendrite) grouped dendrites had 16.1±4.083 (Mean±SEM, n = 4 fields, 193 crossings, total 1041 µm dendrite). This difference was statistically significant, with p = 0.0016 (t = 4.038, df = 12). Hence, it seems that neighboring dendrites in assembly have a tendency to enrich the crossing rates of their branches.

### A New Approach for Representation of Dendritic Networks’ Structure and Interaction with Axonal Networks

The high incidence of crossings among neighboring dendrites suggests their structural overlap to be a regulated phenomenon. However, manual analysis of this behavior, as presented in Figure 2, is overwhelmingly tedious and slow.

Current computational tools for analysis of neuritic structures are based on reconstruction of single-cell neuroanatomy of which three different reconstruction approaches exist. The first uses skeletonization algorithms where the neurite image is binarized and thinned down to the neurite thickness [36], [37]. The second approach traces neurites as a series of interconnected vectorial cylinders, each represented by diameter, spatial coordinates and the connectivity to other cylinders in the tree [38]–[41]. The third approach, used mainly in serial reconstructions of electron microscopy, represents neuritic morphology by three-dimensional reconstruction of membrane surface [42], [43]. These three approaches accumulate a vast amount of information about the neurites’ geometry, enabling the measurement of various neuritic arborizations parameters [43]. However, a key limitation of these approaches is that while they condense neuromorphological data generated from single cells, they can not account for environmental influence on neurite morphology.

Based on our findings that neighboring dendrites control the crossing of their branches, we adopted a novel reconstruction approach. We developed a MATLAB-based program, named “Crossing Connectivity Mapping” (CCM), which maps dendritic networks by identifying the intersections between dendritic branches and reconstructing their topology. It recognizes intersections since they are wider than the branch segments composing them (Figs. 1A1, 1A2, 1A3). The intersections are then labeled, centered by a single point and connected by lines to other contacts with which they share branch segments (Fig. 1A4. 1A2, 1A3, 1A4, 1A5, 1A6, 1A7). This approach obviates single cell labeling and provides data on neurite networking not detected by previous reconstructing approaches, such as:

1. Geometrical properties of the structural overlap among dendritic trees, including their positioning, crossing and proximity between sister and non-sister branches.
2. Identification of hidden organizations of dendritic subnetworks selected by various geometrical properties of the dendritic branches and their crossings.
3. Morphological data on the structural interactions between dendritic and axonal networks, based on morphometrical quantification of their intersection.

### Dendritic Branches Greater than 1 µm in Caliber Exhibit Favored Intersection Angles

**Figure 3:**
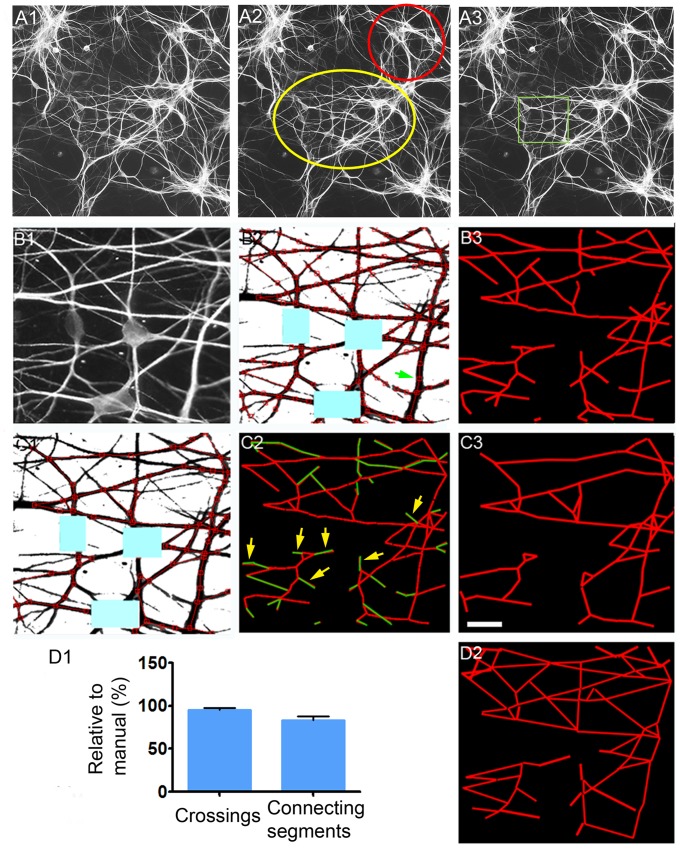
Reconstruction of dendritic networks and sub-networks by CCM. (A) An area in a relatively dense one week old hippocampal culture (A1, MAP2) has ordered (yellow ellipse in (A2)) and less ordered (red circle in (A2)) regions. (A3) Within the ordered area, an even more highly organized region was found (green square) and used for analysis in (B). (B) Dendritic network reconstruction using CCM. (B1) High magnification of the boxed area in (A3). (B2) Reconstruction of (B1). (B3) Connectivity map of (B2). (C) Reconstruction of a sub-network of >1 micron caliber branches. (C1) Reconstructed “thick” branches network. Few thick branches adjacent to the cell bodies were excluded due to deletion of their crossings (located within the blue rectangles). (C2) The connectivity map of the “thick” (red) and fine (green) branches. Yellow arrows show that a large portion of the thin dendrites are at the edges of growing branches. (C3) The “thick” branches sub-network’s connectivity. (D) Comparison between CCM (B3) and the manual reconstructions (D2). (D1) The efficiency of junction and connecting segments detection by CCM compared to that of manual analysis (100%). Shown is the mean ± SEM. Scale (in C3): A - 60 µm; B–D - 15 µm.

A unique advantage of the CCM program is that it takes into consideration the dendritic branches’ caliber and thereby may reveal hidden patterns of organization within seemingly disorganized dendritic networks. An example is shown in figure 3A1, where a field of a 12 day old culture appears as a grouping of dendrites apparently lacking organization. However, a deeper observation revealed varying levels of organization, with some areas having apparent disorder (Fig. 3A2, red circle) and others had slightly more parallel branch positioning (yellow circle). A sub-field within the yellow marked area (green square, Fig. 3A3) displayed an even higher abundance of parallel branch positioning, as shown in figure 3B1. The field was analyzed by CCM (Fig. 3B2), yielding the reconstructed mesh shown in figure 3B3. A second analysis included branches larger than 1 µm in caliber (Fig. 3C1). The reconstructed sub-network is presented both with and without the thinner branches (Figs. 3C2 and 3C3).

Next, the efficiency of the CCM reconstruction was evaluated in six fields (Fig. 3D1), by comparing it to that of manual detection (shown in Fig. 3D2). CCM was found to be highly effective, detecting 95.5%±0.803 of dendritic crossings (Mean±SEM, n = 5 fields, 132 crossings) and 83.52%±1.705 of dendritic segments (Mean±SEM, n = 5, 164 segments) detected manually.

**Figure 4:**
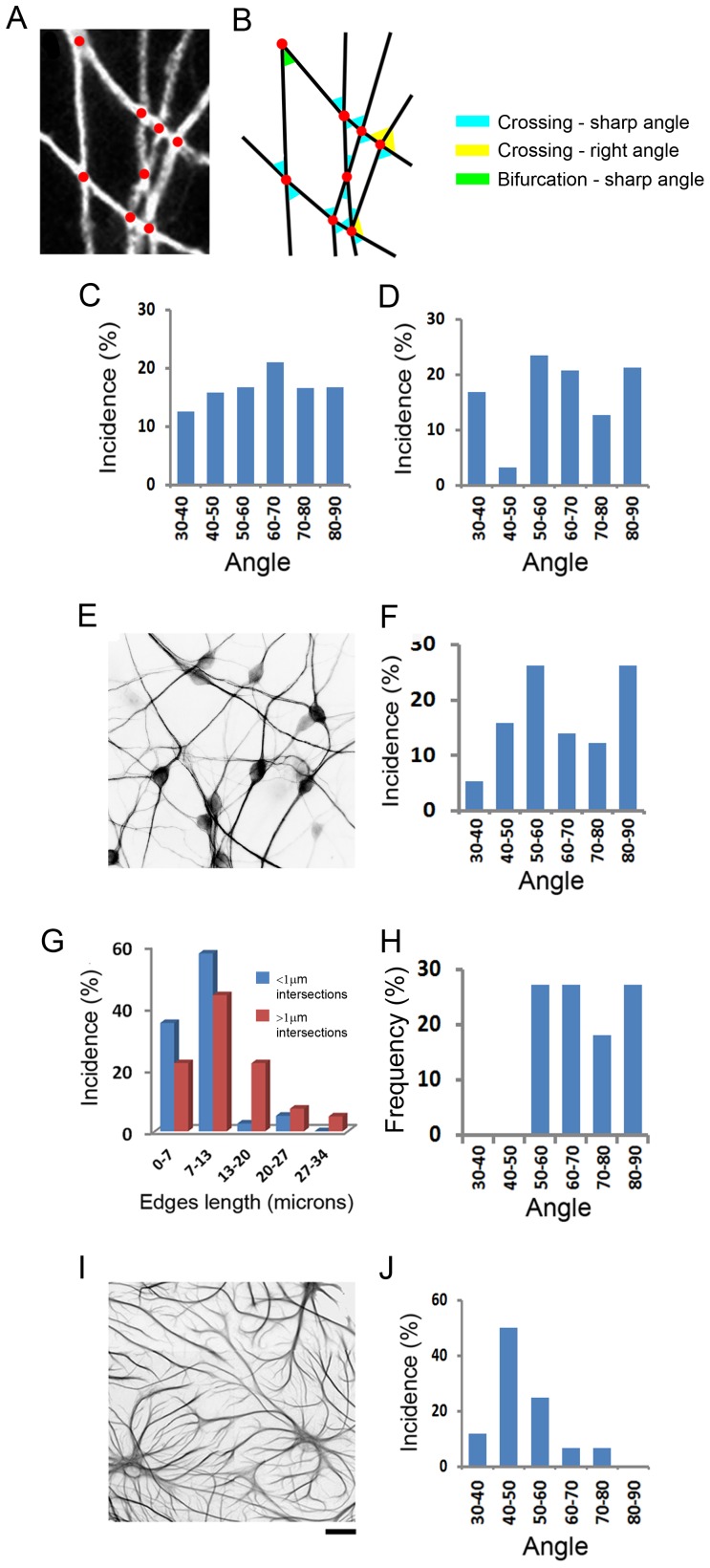
Dendritic branches cross through preferable angles. (A, B) The procedure for analyzing dendritic crossing angles. A mesh of crossing dendritic branches (A) is reconstructed in (B) based on the intersections location (red spots) and their connections (black lines). Acute and right angles between crossing and bifurcating branches are collected. (C, D) Analysis of the field shown in figure 3D. (C) Crossing angle distribution of combined fine/thick branches. (D) Distribution of angles among >1 micron caliber branches. (E, F) A dendritic network (E) with similar preference of crossing angles ranges among “thick” branches as in (D) but of higher incidence (F). (G) Length distribution of fine (blue bars) and “thick” (red bars) intersections from the field in figure 3D. (H) Frequency of crossing angle range selection among 15 dendritic networks. (I) An astrocytic network. (J) Intersection angle distribution of (I). Note the 40°–50° range preference, which is absent from the dendritic networks (H). Scale (under H): A –4 µm; F, I –20 µm.

The first morphometric parameter to be analyzed was the distribution of branch-to-branch intersection angles. This distribution can reveal existence of favored crossing orientations, as a sign of regulated organization of the dendritic network. Intersection angles were those located between crossing or bifurcating branches (Figs. 4A and 4B). Two crossing angle types were excluded from the analysis, according to the following selection criteria:

1. *Blunt vertex angles:* Most crossings were between two straight branches, producing two complementary pairs of blunt and sharp vertex angles. This data redundancy was overcome by including only the sharp edges (Fig. 4B, blue) in the analysis. Other types of angle crossings, like in bifurcations and non-vertex crossings were included, except if they were >90°.
2. *False scanning conditions-derived crossing angles:* Under certain conditions CCM’s scanning misdetected crossing and connecting segments within shafts of very thick branches (an example is shown with a green arrow in Fig. 3B2). Crossing angles produced within the shafts were usually small and were excluded by restricting the analysis to angles >30°. Although the frequency of within-shaft crossings and connections are tunable and could be reduced, their presence was actually advantageous. They enabled identification of single-contact connections found mainly at the tip of growing dendrites (see yellow arrows in Fig. 3C2), and at the boundary of the working field.

Analysis was first performed on the network in figure 3B1. When fine (<1 µm caliber) and thicker branches were analyzed in combination, crossing angle distribution was relatively homogenous with a slight prevalence of the 60°–70° range, which represented 20% of crossings (Fig. 4C). By contrast, among “thick” (>1 µm ) dendritic branches, dendritic crossings concentrated in 3 ranges: 50°–60°, 60°–70° and 80°–90°, at much higher preponderance (57%, 14% and 77% more, respectively) than resulted from analysis of all dendrite branches together (Fig. 4D). Each one of these ranges accounted for 20% or more of dendritic crossings, the threshold value chosen for favored crossing angles. Another example of a field with ordered thick dendritic branch organization of a similar crossing angle distribution is shown in figures 4e and 4F, peaking at 50°–60° and 80°–90° ranges. These findings suggest that orientation of the thicker dendritic branches is under more stringent regulation than that of fine branches. It is therefore likely that a network of thick dendritic branches (>1 µm) will form fewer intersections than will thinner dendrites. Accordingly, the distances between thick dendritic intersections where found to be larger than those of the fine branches (Fig. 4G).

In order to verify these results two approaches were taken:

First, dendritic intersection angle distribution was compared across 15 different fields. In each field, intersection angle range that surpassed a 22% prevalence threshold was registered and measurements from all fields were represented in Fig. 4H. Specific intersection angle ranges passed the prevalence threshold (50°–70° (>50%) and 80°–90° (27%), whereas crossing angles at 30°–50° in all fields never passed the threshold.

Secondly, a different type of network was investigated, that of astrocytic processes (Fig. 4I). The results show that order, bearing different characteristics, appeared among the astrocytes. Astrocytes crossed 50% of their processes in an angle range of 40°–50° (Fig. 4J). The fact that astrocytes prefer a range of crossing angles that is rarely occupied by crossing dendrites indicates a distinctive organization of the two cell populations, and ensures reliability of the analysis. Notably, neurons exhibited 5 times more intersections than did astrocytes. This difference can be perceived in contrast to dendrites (Figs. 4H), astrocytic processes tended to grow in tiling-like configurations, avoiding contact (Fig. 4I). These findings strengthen our previous suggestion that dendrites actively generate crossings.

### Orientation of Axons Traversing Dendritic Intersections is Influenced by the Intersection’s Angle

**Figure 5:**
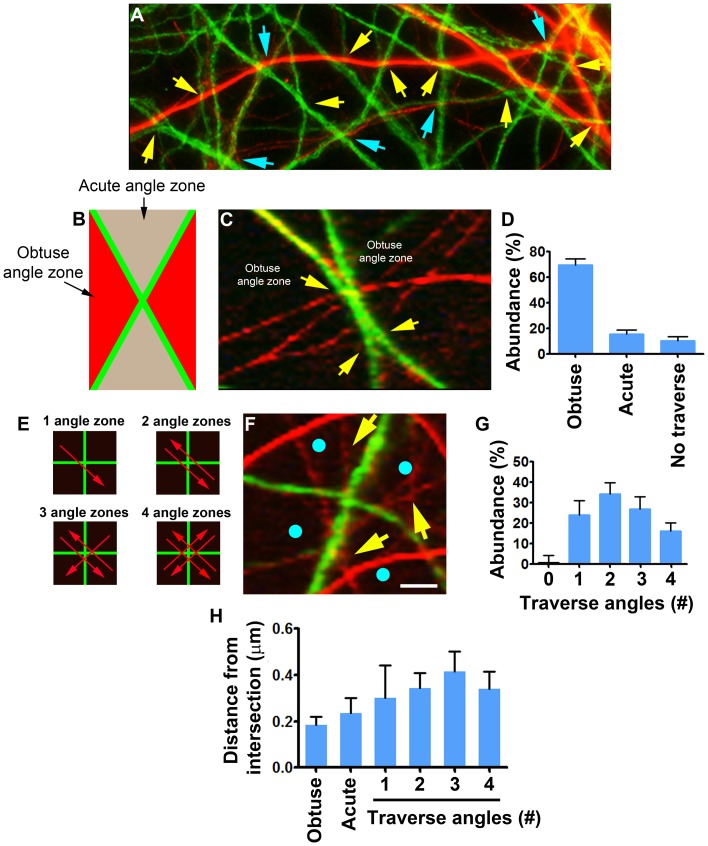
Orientation of axonal traverse near dendritic intersections is affected by the dendritic crossing angle. Green = dendrites (MAP2); Red = axons (NFM). (A) Axons tend to grow toward the vicinity of dendro-dendritic intersections. Shown are single axons traversing multiple dendritic intersections at angles 80°–90° (blue arrows) and non-80°–90° (yellow arrows). (B) Definition of obtuse (red) and acute (gray) angles of a non-80°–90° crossing between dendritic branches (green). (C) Axons traversing a non-80°–90° dendritic branch crossing. Traverse orientation is restricted to the obtuse angles zones. Axo-dendritic contacts are indicated by arrows. (D) Quantification of the occurrence of axonal traverse of non-80°–90° crossing through the obtuse and acute angles. (E) 80°–90° dendritic crossing (green) traversed (red arrows) in various combinations of its four angle zones (black). (F) Axons traversing an 80°–90° angle dendritic branch crossing (yellow arrows) from 3 out of 4 angle zones (blue spots). (G) Quantification of the occurrence of axonal traverse of 80°–90° crossings through the 4 possible angle zones. Intersections which were not traversed were indicated on the X axes as “0”. (H) Quantification of the distances of the traversing contacts from the dendritic intersections. Scale: A-35 µm, B, C–5 µm.

Crossing among dendritic branches through preferable angles influenced not only dendritic morphology, but also the interactions of dendrites with axons. It was observed that axons tended to grow toward vicinities of dendritic intersections (Fig. 5A), traversing 97.3% of them (n = 277 intersections of 21 fields) at a distance of 2 µm or less. Interestingly, the orientations of axons when traversing near the intersections were dependent on the dendritic crossing angle. Figures 5B, 5C, 5D show that in non-80°–90° intersections 69.6%±4.6 of the axons traversed through the obtuse angle, whereas only 15.6%±3.2 through the acute angle (mean ± SEM, n = 207 crossings from 21 fields, 4 experiments, p<0.0001 One way ANOVA). By contrast, axons traversing 90° dendritic intersections oriented through each of the four possible angles, or their combinations (Figs. 5E and 5F), in approximately similar frequencies (Fig. 5G) (p = 0.1997, one way ANOVA, n = 70 intersections of 21 fields, 4 experiments). Regardless of these orientation distinctions, the distance of axonal traverse sites from both intersection types was similar, ranging from 0 to 2 µm and averaging between 0.19±0.03 µm to 0.42±0.08 µm (mean ± SEM), for non-80°–90° and the 90° crossing respectively, with no statistical difference (p = 0.3716, one way ANOVA) (Fig. 5H).

### Axons Preferentially Transverse Dendrites in Proximity to Dendritic Intersections

**Figure 6:**
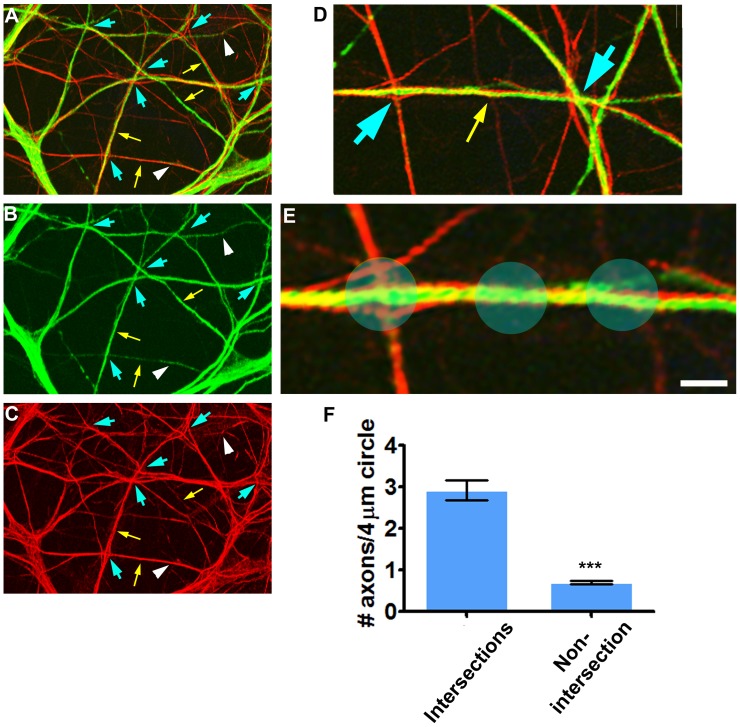
Axons traverse dendrites preferably near dendritic intersections. (A–C): The same field seen as merge (A), dendrites (MAP2) (B) and axons (C). Axons traverse predominantly near dendritic intersections (blue arrows) and rarely at non-intersecting regions along dendrites (white arrowhead). Most non-intersecting shafts of dendritic branches are not traversed but rather fasciculated by axons (yellow arrows). (D) Higher magnification of the central region of (A) shows traverse events near (blue arrows) and at a distance (yellow arrow) from dendritic intersections. (E) Further magnification of the left region of (D): Blue scanning circles (4 µm diameter) used for quantification of traverse frequency. (F) Traverse frequency was considered as the number of axons located within the circles. Scale: A–C - 30 µm; D - 13 µm; E - 3 µm.

Such close apposition of axonal traverse sites and dendritic intersections hinted that axons grow preferentially towards dendritic intersections. We previously presented initial documentation of such phenomenon [32], yet there remained a need for its quantification. Especially, we were interested to understand if intersection-oriented growth in axonal networks results in convergence of multiple axons to vicinities of single dendritic intersections. Such a scenario may produce and localize synaptic clustering. Thus, we compared the incidence of axo-dendritic contacts near dendritic intersections to that occurring in non-intersecting regions along dendrites. As shown in figures 6A, 6B, 6C, 6D, axonal traverse took place mostly at or within two microns of dendritic intersections. This was measured by counting the number of axons found within a 4 µm (blue scanning circles) centralized on intersections or dendritic shafts (Fig. 6E). It was found that at 0–2 µm distance from intersections, the number of transversing axons ranged between 0 to 6, averaging at 2.92±0.23 (mean±SEM, n = 50, 5 fields, 3 experiments) (Fig. 6F). At longer distances from dendritic intersections, the number ranged from 0 to 3 and averaged approximately 4 fold lower at 0.71±0.05 (mean±SEM, n = 283, 5 fields each from 3 experiments, p>0.0001, two-tailed *t test*).

**Figure 7:**
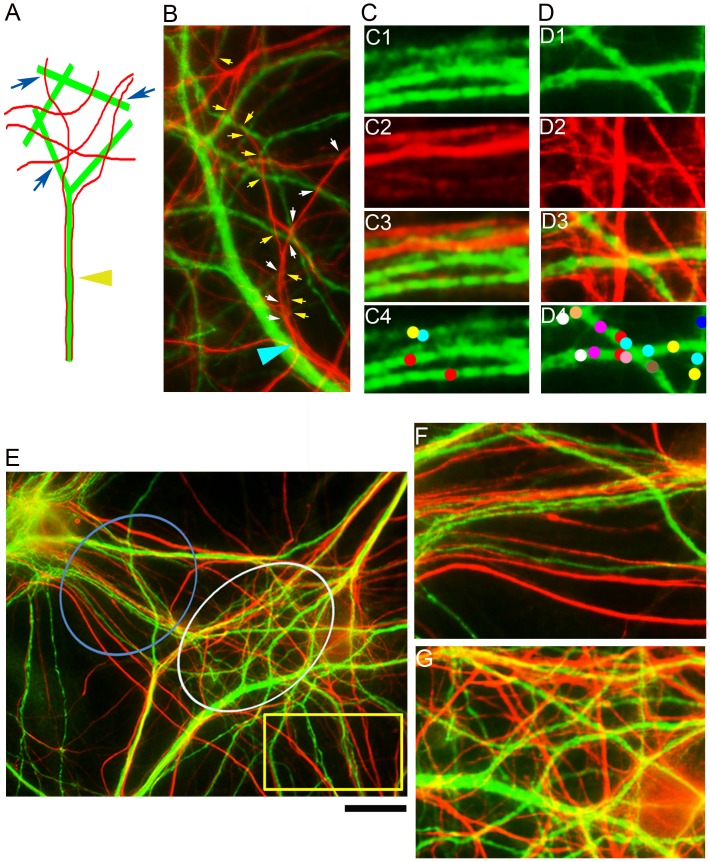
Axons preferentially traverse heavily intersecting dendritic branches. Green = dendrites (MAP2); Red = axons (NFM). (A) Axons tend to fasciculate (yellow arrow) with dendritic branches at areas of low dendritic branch-to-branch crossing frequency, but prefer to traverse (blue arrows) regions with high rates of dendritic crossing. (B) Two axons defsciculate (blue arrow) from a thick, rarely-crossing dendritic branch to traverse several highly intersected dendritic branches (arrow colors specific to individual axons). (C) Non-crossing dendritic branches (C1) and axons (C2) interact (C3), forming only few axon-dendrite contacts (arrow colors specific to individual axons). (D) When crossing dendritic branches (D1) and axons (D2) interact, the axons heavily traverse dendritic branches (D3), forming axo-dendritic contacts involving multiple axons (D4). (E) A dendritic hub-like organization. The center of the hub (white circle) is composed of heavily crossing branches whereas branches located at the hub’s periphery (blue and yellow marks) have fewer axonal crossing events. (F) Magnification of the yellow rectangle region in (E) shows alignment of axons with rarely crossing dendritic branches and low frequency of axo-dendritic contacts. (G) Magnification of the white circled hub’s center in (E) showing high frequency of dendro-dendritic and axo-dendritic contacts. Scale (black line underneath (E): B - 15 µm; C, D - 6 µm; E - 30 µm; F, G - 10 µm.

The higher incidence of axonal traverse near dendritic intersections suggested that dendritic crossings are favored traverse targets for axons. To test this hypothesis, we compared axonal traverse frequency in areas of heavy and rare dendritic crossings. Axons aligned or fasciculated with non-intersecting dendritic regions, whereas they predominantly traversed intersecting regions, as illustrated in figure 7A. At the fasciculation area, each axon contacted a single dendritic branch, whereas in crossing areas axons contacted multiple closely associated dendritic branches by traversing them (Fig. 7B). As a result, traverse contact density was higher at crossing regions than non-crossing. This is exemplified in figures 7C and 7D, where axons associated with non-crossing dendritic bundles formed 1 traverse site per 20 µm dendrite, whereas with crossing dendritic branches they formed 5 fold more.

In order to quantify this phenomenon at a large scale we focused on a hub-like dendritic organizations found in the culture (Fig. 7E), where the above axonal behavior was most evident. Dendritic hubs were composed of peripheral regions of non-intersecting dendrites (blue circle and yellow rectangle) connected by a center of heavily crossing dendritic branches (white circle) (Fig. 7E). While dendritic branches in the periphery where rarely traversed by axons (Fig. 7F), the crossing branches in the center were heavily traversed (Fig. 7G).

**Figure 8:**
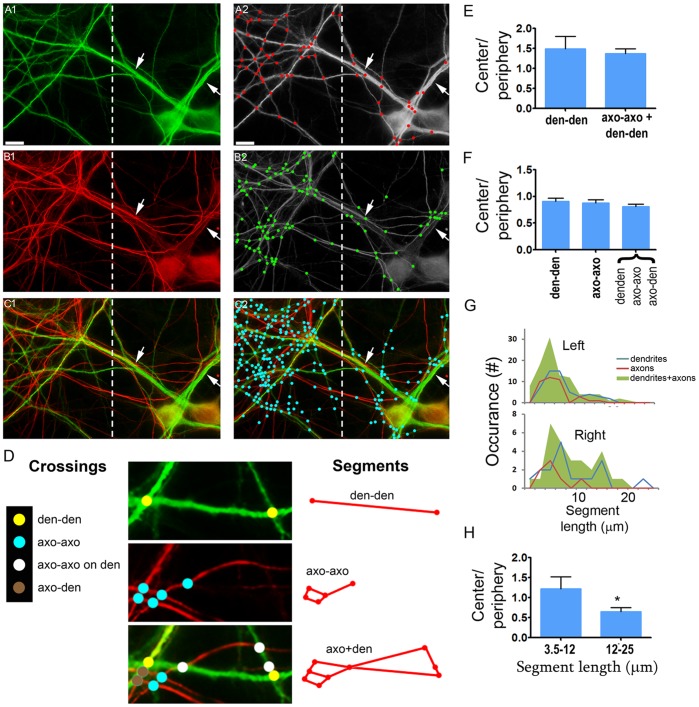
Quantification of preferential axonal traverse of heavily crossing dendritic branches. *I. definition of high vs low frequency crossing regions:* (A1–C2) A field which is part of a dendritic hub. (green = dendrites (MAP2), red = axons (NFM)). (A1) The field was divided into two regions: left (“center”) – frequently crossing branches; Right (“periphery”) – rarely crossing branches including dendritic stems (arrows). The crossing points are shown by red spots in (A2). (B1, B2) axons and their crossing points in green (B2). (C1, C2) merge of A1/B1 and A2/B2. Note the higher crossing frequency in the center region compared to the periphery in all cases. *II. Nomenclature of the various crossing types (D, left):* den-den = crossing points between two dendritic branches; axo-axo = crossing points between two axons; axo-axo on dendrite = axo-axo contacts aligned with den-den contacts; axo-den = contacts between axons and a dendritic branchs. Examples are shown in the middle panels. *III. Defining the crossing connecting segment types (titled ‘segments’):* den-den = between two den-den crossing points; axo-axo = between two axo-axo crossing points; axo+den = between all crossing points in a merge image. *IV. Analysis:* (E) Crossing density ratio (at hubs’ center vs periphery), normalized to dendritic length. (F) Ratio of the average length of neuritic segments connecting the crossing points. E and F are combined analysis of 4 hubs. Shown is the mean ± SEM. (G) Length distribution of the connecting segments of the field shown in panels A–C. (H) The segment length distribution in (G) was narrowed to two subgroups. Y axis presents the values of right/left ratio of [#segments (axo+den)/(axo-axo+den-den)]. Shown is the mean ± SEM. Scale: A1–C2 - 7 µm; D, middle panels - 3 µm.

CCM was used to analyze separate and superimposed images of dendrites and axons in the aforementioned dendritic hubs. Hubs appropriate for CCM analysis were regions having distinct center and periphery shapes and <2.5/10 µm^2^ intersection density, as in that shown in figures 8A, 8B, 8C. To this end, a nomenclature of the various types of intersections and connecting segments of the neural networks was established (Fig. 8D). Then, the ratio of dendritic branch intersection density was compared between the hub’s center and periphery. It was found to be significantly higher that 1 (p<0.0001, F test), reaching a value of 1.488±0.31 (Mean ± SEM, n = 4 hubs, 193 crossings, 1707 µm dendrite) (Fig. 8E). When the axonal network was superimposed on the dendritic one the center/periphery crossing density ratio was conserved (p = 0.7419, unpaired two-tailed student *t* test) at a value of 1.373±0.1143 (Mean ± SEM, n = 4; 511 crossings (den-den, axo-axo, axo-den)) (Fig. 8E). Apparently, higher crossing density at the hubs’ center would require their connecting segments to be shorter than those in the hub’s periphery. Indeed, the ratio of dendrite segment length between the center and periphery in dendritic, axonal and mixed axonal-dendritic networks where smaller than 1 (p<0.0001, F test) (Fig. 8F). They reached values of 0.91±0.056 (den-den), 0.88±055 (axo-axo), and 0.81±049 (all types) (Mean ± SEM, n = 4 hubs; 355 segments of all types) and were statistically similar (p = 0.8522, one-way ANOVA, Bonferroni’s test). These results were verified by plotting the length distribution of dendritic segments of the field in figure 8A1. (Fig. 8G). The lengths value at the hub’s centers skewed toward smaller values compared to those in the periphery. This difference was quantified by narrowing the distribution to two ranges, 3.5–12 µm and 12–25 µm, and comparing the number of segments (axo+den)/(axo-axo+den-den) for each range between center and periphery (Fig. 8H). The value for the 3.5–12-µm group was more than 20% (1.223±0.15) higher in the center than the periphery, whereas for the 12–25 µm group it was smaller than the periphery by close to 40% (0.6457±0.052), (Mean ± SEM, n = 4 hubs, 676 segments). These values of the two segments length groups were statistically different (p = 0.0111, two tailed student *t test*, df = 6).

### Axons Cluster Synaptic Connections at the Vicinity of Dendritic Branch Intersections

**Figure 9:**
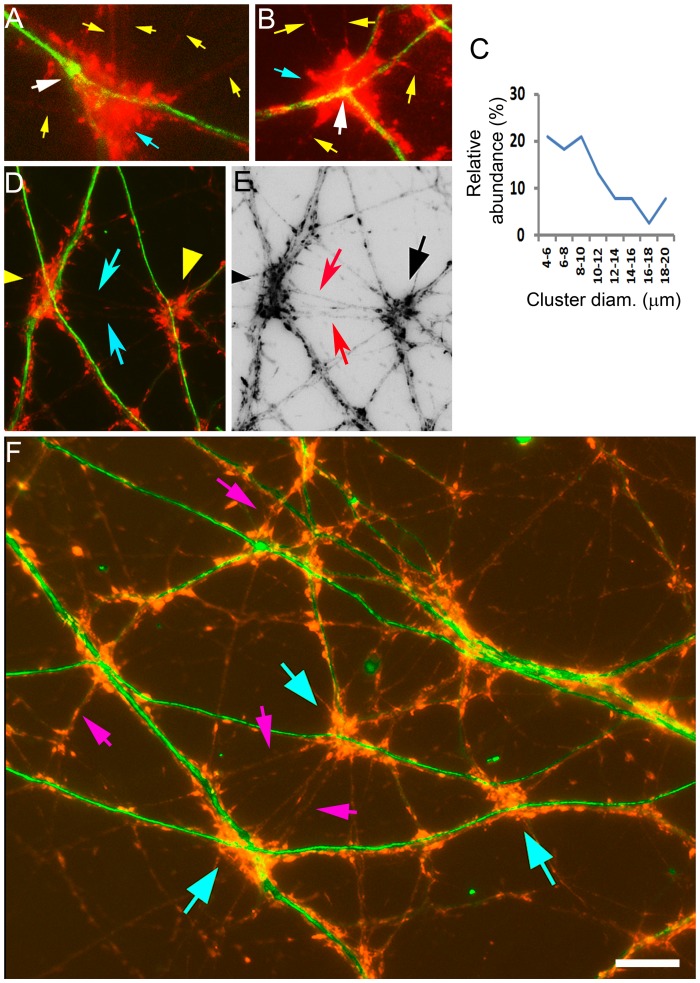
Axons cluster their synapses near dendro-dendritic crossings by converging predominantly onto these sites. Green = dendrites (MAP2); red = active synapses (FM1-43). (A) A non-90° dendritic intersection contacted by 7 axons (yellow arrows), most of them traversing through the obtuse angles zones. The axons cluster their active terminals (blue arrow) near the intersection (white arrow). (B) A 90° dendritic intersection traversed by axons from three different zones (yellow arrows), associated with a cluster of active axonal terminals (blue arrow). (C) Size distribution of synaptic clusters located at dendritic intersection vicinities. (D) Synaptic clusters on two dendritic intersections linked by multiple axons (arrows). (E) Same regions as (D) showing an inverted image of dendritic clusters and connecting axons (arrows). (F) A network with active synapses localized to dendritic intersections (arrows). Most clusters are linked through bundles of multiple axons(examples are indicated by pink arrows). Scale: A, B - 10 µm; D - F20 µm.

The results above revealed a tendency of axons to not only traverse crossing dendritic branches but to also converge near their intersections. As a result, dendritic intersection vicinities were enriched with axo-dendritic contacts (Fig. 6) These contacts were capable of uptaking and releasing the synaptic vesicle recycling dye FM1-43 [44] (Fig. 9A), an indication that they bear active synaptic connections. Thus, dendritic intersections were associated with clusters of active synaptic connections (Figs. 9A and 9B). The diameter of these clusters ranged between 4–20 µm, where 41% of them surpassed 10 µm diameter (Fig. 9C), enough to contain dozens of synaptic connections. It was also observed that synaptic clusters on different dendritic intersections could be linked by several traversing axons (Figs. 9D and 9E). Such growth of axons through multiple dendritic intersections produced, eventually, an ordered appearance of the culture where synaptic clusters were allocated according to the map of dendritic crossings (Fig. 9F).

## Discussion

This work presents a new link between dendritic morphogenesis and axonal wiring. It demonstrates that neighboring dendrites cross their branches in preferred angles and that this behavior causes axons to navigate toward these crossings and converge near their intersections. Eventually, such neuritic interactions results in the accumulation of synaptic clusters in the vicinity of dendritic intersections. This structure-function link may serve as a mechanism for efficient network wiring, as well as for synaptic co-activity and plasticity.

### Dendritic Morphogenesis is Underlined by Coordinated Inter-dendrite Branch Growth and Crossing

The results shown in figures 2, 3, 4 imply that dendritic morphogenesis is deterministic. Dendrites tend to cross their branches with those of neighboring dendrites rather than to sprout them randomly (Fig. 2). Moreover, they regulate their crossing angles, displaying preference of the angle ranges 50°–70° and 80°–90° (Figs. 3 and 4). Such controlled crossing means that dendritic branches grow in relation to the spatial positioning of their neighboring branches, such that the direction in which they grow is determined by their proximity and position relative to neighboring branches. Greater proximity to a neighboring dendritic branch increased the chance of intersection. The orientation it would have to select in order to cross through one of the two preferable angle ranges depends on its relative positioning to the neighboring branch prior to crossing, and the length it would need to extend would be directly related to its distance from the neighbor. However, when a branch faces multiple neighbors, its growth orientation is a function of its position relative to all neighbors. Such a neighbor-related growth mechanism may be relevant to neural activities that involve dendritic structural modifications, such as brain development [45], plasticity [46] and cognition [47].

This kind of directed dendritic growth contradicts other studies suggesting that dendritic morphology develops randomly. Previous experimental [48]–[50] and computational [51] works studied dendritic trees as individuals and not as part of the dendritic assembly. Dendritic trees display enormous structural diversity which justifiably can be seen as random. However, this high structural diversity may arise from local non-random sprouting of branches, such as through neighbor-related mechanisms. For instance, when each branch of a dendritic tree grows in a particular direction, dictated by its local environment, the ramification of the entire tree would eventually be distinct from that of other trees whose branches experience different surroundings. Thus, our results suggest that despite their structural diversity, dendritic trees in neuronal networks share a common morphogenetic mechanism - a neighbor-related growth of branches. The structural diversity which emerges from this mechanism yields dendrites growing in neighborhoods of branches varying in their spatial configurations.

Noteworthy is that this neighbor-related mechanism was favored by thick (>1 µm) but not by thin branch segments (Figs. 3, 4). Thin segments appeared mostly near growing tips are therefore likely to be young growing branches heavily involved in target search. This search demands a high degree of freedom in growth directions, which may explain the lack of preferred crossing angles by the thin segments. In contrast, thick branch segments, especially those farthest from the growing tips, showed clearly regulated growth direction. Thus, it is possible that developing dendritic trees’ formation and extension of new branches occurs randomly, whilemature branches are organized according to the spatial configuration map of their neighbors.

### Dendritic Crossing-related Wiring Shapes Axonal Wiring Topology

**Figure 10:**
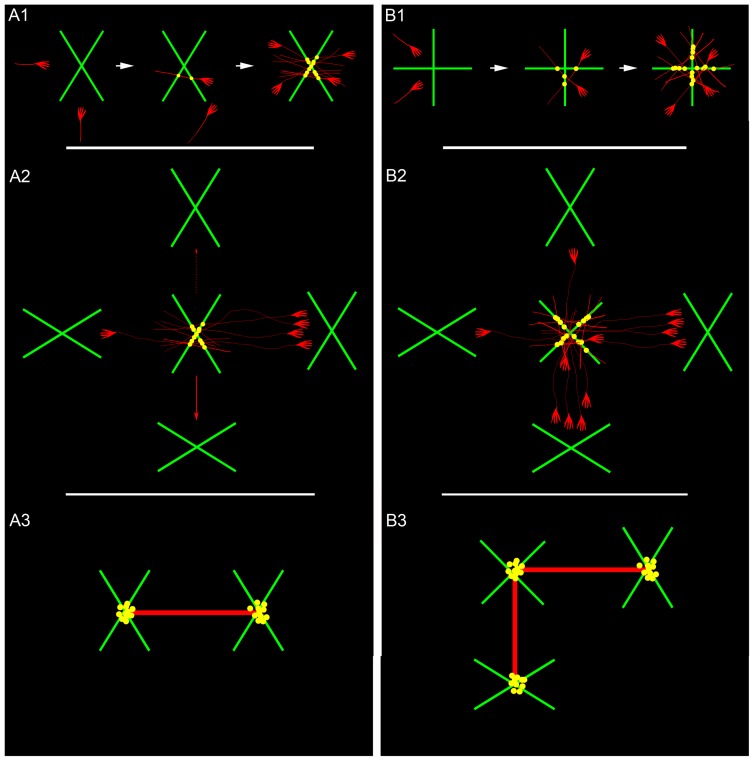
The influence of angle and position of dendritic branch crossings on network wiring topology and synaptic clustering. Green = dendrites; Red = axons. Left panels – wiring through a 60° dendritic intersection: (A1) (left) Two axons reaching a dendritic branch crossing from the blunt and acute angles zones. (Middle) Only the axon in the blunt zone traverses near the crossing site, making two contacts, one on each branch (yellow spots). (Right) Additional axons traversing in the same way align in an array, producing multiple contacts at the dendritic intersection vicinity (yellow dots). (A2) Four 60° angle crossings are positioned against the four angle zones of the central crossing taken from (A1). From the central crossing towards the periphery, the configurations are: obtuse to obtuse (center to right), obtuse to acute (center to left), acute to obtuse (red line pointing down), and acute to acute (dashed arrow). Based on the axonal preference to traverse through obtuse angles, the obtuse to obtuse configuration will become linked by the axonal group. Other crossings will be weakly (obtuse to acute) or rarely (red arrows) linked. (A3) The (A2) configuration yields central and the right crossings that become wired and both bear synaptic clusters. Right panels – wiring through a 90° intersection: (B1) Axons approaching a 90° intersection can traverse through the four angle zones. (B2) Same configurations as (A2) but with a 90° crossing at the center. Due to the higher traverse freedom that axons have with the 90° intersection, they can link two crossings in this configuration. (B3) The outcome is three wired crossings, all bearing synaptic clusters.

Figure 10 illustrates the possible formation and outcome of a dendritic crossing-dependent wiring mechanism. Thick dendritic branches (>1 µm width) preferably cross their neighbors in two angle ranges, 50°–70° and 80°–90° (Fig. 10A1 and 10B1). Axons encountering the 50°–70° crossings prefer to traverse them through their obtuse angles and only rarely through the acute ones (Fig. 10A1). When such patterns are mimicked by additional axons, they align at the obtuse angle’s area and group their contacts near the dendritic intersection (yellow dots, Fig. 10A1).

These axons can then continue to grow and traverse additional dendritic crossings. If the additional crossings are of the 50°–70° type, as exemplified in figure 10A2, the axons will preferentially traverse those whose obtuse angles are aligned with the axon’s growth path (Fig. 10A2). Any other alignment combination may result in little or no traverse incidence. When axons traverse the new dendritic intersection, they form a synaptic cluster at the intersection vicinity. Following this wiring principle, in the particular dendritic organization illustrated in figure 10A2, two out of five dendritic crossings connect through multiple axons and possess clusters of active synaptic connections (Fig. 10A3).

By contrast, dendritic crossings in the angle range of 80°–90° provide axons with higher degree of traverse orientation possibilities than do the 50°–70° crossings (Fig. 10B1). As a result, under the same dendritic configuration as in figure A2, but with a 90° crossing at the center, 3 instead of 2 dendritic crossings are transversed by axons and have synaptic clusters near their intersections (Figs. 10B2 and 10B3).

The networks in figures 10A3 and 10B3 are distinct in terms of both wiring topology and of their patterns of synaptic clustering. These distinctions may be attributed to axonal reaction, in terms of growth directionality, to the angles and orientations of dendritic branches intersections.

According to this dendritic crossing-dependent wiring model, axons navigate through adjacent dendritic intersections, such that axonal path and targets are defined by the location, density and angle of dendritic intersections. As a result, the dendritic branch intersection network dictates the axonal growth patterns and subsequent clustering of synaptic inputs, forming a direct link between morphology and physiology.

### Dendritic Crossing-dependent Wiring may Derive from the “Minimal Wiring Length” Principle

Presently, we describe an additional aspect of the relationship between dendritic branch crossing and axonal traverse. Axons preferentially traverse through the vicinity of dendro-dendritic intersections (Fig. 6). It appears that wiring-cost logic may explain such axonal preference. The proximity between two crossing dendritic branches is highest near and at their intersection. By reaching this area, axons require only short lengths to contact both target branches, satisfying the “minimal wiring length” principle to optimize wiring efficiency in the brain [31]. Hence, axonal traverse near or at dendro-dendritic intersections is a means for efficient axonal wiring.

### Axonal Convergence onto Dendro-dendritic Intersections – a Mechanism of Synaptic Clustering

Out of the various mechanisms that were suggested to underlie synaptic clustering mentioned in the introduction, our results implicate axonal convergence [9], [14], [15]. The accumulation of synapses into clusters in the vicinity of dendritic branch intersections is possibly due to the high number of axons converging at this site. However, there is still a possibility that the clustering resulted from a localized enhancement in synaptogenesis, perhaps via a plasticity-related mechanism. There is some evidence to support this suggestion, as we and others reported that clustered synapses are stronger than are isolated synapses [34], [52] and that strong synaptic connections are more clustered than are weak ones in the cortex [53]. It remains to be determined if synaptic clustering at the crossing sites is due to an accumulation of axo-dendritic contacts made by the converging axons, or whether it is the result of elevated axonal terminals density, specifically near the dendro-dendritic intersection. Both options raise the possibility that dendro-denritic crossings are preferable sites for synaptic clustering, perhaps by actively attracting axons and/or stimulating local synaptogenesis.

Regardless of the underlying synaptogenic mechanism, synaptic clusters at the dendritic intersections are large enough to contain several active axonal terminals originating from the same axon. The close proximity of such sister synapses increases the likelihood that they can be co-activated, as seen elsewhere regarding synaptic inputs on dendrites of the developing hippocampus [22]. At the same time, the convergence of multiple independant axons to single crossing sites and the large dimensions of the cluster they form bring such non-sister synapses into tight association, potentially resulting in synchronized activity. If one or both of these options are correct, than a new structure-function link can be drawn: crossing between dendritic branches may synchronize synaptic firing among axons because the dendritic intersection is a preferred sites for axons to converge and cluster their inputs. This link can be meaningful even at the scale of entire neuronal networks, depending on the crossings’ density. High crossing densities are likely to be found when overlap among dendritic trees is extensive, as found in the center of dendritic hubs in culture (Figs. 6–8), and the opposite was found in networks of non-overlapping dendritic fields [1]. Hence, we postulate that the extent of dendritic-branch-to-branch crossings can tune and shape the pattern of activity synchronization in neuronal networks.

### Broader Applicability of the Intersection Connectivity Mapping Approach

The organization of dendritic branches, and particularly their interaction with axons, is extremely complex, even in a relatively simplified cell culture neuronal network model. The analysis of this complexity was made possible by the CCM software, which not only provided the means to describe and quantify complex neuritic meshes, but also exposed hidden network organizations, such as that of the thick dendrite sub-networks. This unique capability opens new possibilities for the discovery and investigation of heretofore unknown structural and wiring architectures of neuronal networks. We anticipate that CCM should reveal structural properties of larger networks and perhaps even 3D neuronal networks. Beyond that, the analytical approach of CCM can be expanded to study overlapping of other, nonneuronal networks.
